# Synergistic activity of Ginsenoside Rh2 with *β*-lactam antibiotics and its mechanism of action against methicillin-resistant *Staphylococcus aureus*

**DOI:** 10.1128/spectrum.00547-26

**Published:** 2026-09-28

**Authors:** Ruihuan Chen, Lingyuan Guo, Yimai Deng, Yaoyao Zhuang, Yijie Cheng, Yeqiong Xu

**Affiliations:** 1 Department of Pharmacy, Changshu Hospital Affiliated to Soochow University (Changshu No.1 People’s Hospital) 576492, Changshu, Jiangsu, China; 2 College of Medicine, Yangzhou University 38043, Yangzhou, Jiangsu, China; 3 School of Biological and Food Engineering, Suzhou University of Technology 12583, Changshu, Jiangsu, China; 4 Molecular and Microbial Laboratory, Changshu Institution of Laboratory Medicine, Changshu, Jiangsu, China; Icahn School of Medicine at Mount Sinai, New York, New York, USA

**Keywords:** methicillin-resistant *Staphylococcus aureus*, Ginsenoside Rh2, *β*-lactam antibiotics, synergistic effect, biofilm, two-component system, ATP-binding cassette transporters

## Abstract

Methicillin-resistant *Staphylococcus aureus* (MRSA) presents a significant threat to public health owing to its high pathogenicity and multidrug resistance. The use of antibiotic combinations, including antibiotics and antibiotic adjuvants, has proven effective in treating drug-resistant bacterial infections. This study evaluated the antibacterial effects of Ginsenoside Rh2 (G-Rh2) combined with *β*-lactam antibiotics against MRSA and investigated the underlying mechanisms. Checkerboard assays revealed that G-Rh2 and *β*-lactam antibiotics (cefoxitin, cefazolin, ampicillin, and meropenem) exhibit a synergistic antibacterial effect on MRSA. Bacterial growth curves showed that the combination of 12.5 μg/mL G-Rh2 and sub-MIC *β*-lactam antibiotics effectively inhibited bacterial growth within 24 h. Morphological observation and measurement of intracellular component leakage indicated that the combination of G-Rh2 and cefoxitin (CFX) synergistically compromised the integrity of MRSA cell membranes. G-Rh2 alone had an anti-biofilm effect, and when combined with CFX, it exhibited a synergistic effect. The combination could significantly inhibit the formation of biofilms and eradicate mature biofilms. To elucidate the molecular basis of the synergistic antibacterial effect, transcriptomic analysis was performed. The data demonstrated that the synergy correlates with the downregulation of *vraR* and genes within the *dlt* and *pst* operons. Collectively, our results demonstrate that the combination of G-Rh2 and *β*-lactam antibiotics represents a promising therapeutic strategy against MRSA infections.

**IMPORTANCE:**

Methicillin-resistant *Staphylococcus aureus* (MRSA) is a formidable pathogenic bacterium capable of causing severe infections. This multidrug-resistant organism exhibits resistance to *β*-lactam antibiotics, including penicillins, cephalosporins, and carbapenems. This study reveals that the natural compound Ginsenoside Rh2 (G-Rh2) can restore the susceptibility of MRSA to these antibiotics. When used in combination, G-Rh2 and *β*-lactam antibiotics exhibit synergistic antibacterial activity, leading to compromised cell membrane integrity and disruption of biofilms. Moreover, G-Rh2 significantly delays the development of antibiotic resistance. These findings provide a promising new strategy for using natural products to combat antibiotic-resistant infections, which may extend the lifespan of existing antibiotics and improve the treatment outcomes for patients.

## INTRODUCTION

Methicillin-resistant *Staphylococcus aureus* (MRSA) is a highly pathogenic, multidrug-resistant bacterium. It can colonize healthy individuals and cause severe hospital- and community-acquired infections, such as bacteremia, endocarditis, pneumonia, osteomyelitis, and skin and soft tissue infections (1). *Staphylococcus aureus* was highlighted by the Global Burden of Disease Study 2019 as one of the most impactful bacterial pathogens worldwide, responsible for over 1 million fatalities and 34.5 million disability-adjusted life years (2, 3). The escalating threat of antimicrobial resistance has exacerbated this problem. From 1990 to 2021, the burden of MRSA infections increased substantially in China and worldwide (4, 5). Consequently, MRSA poses a substantial global public health threat.

The resistance of MRSA primarily stems from the expression of the *mecA* gene, which encodes penicillin-binding protein 2a (PBP2a). This altered penicillin-binding protein has reduced affinity for a wide range of *β*-lactam antibiotics, including penicillins, cephalosporins, and carbapenems, and thereby confers MRSA resistance to this important antibiotic class (6). Although antibiotics, such as vancomycin, daptomycin, and linezolid, are used clinically to treat MRSA infections, their utility is constrained by adverse effects and the emergence of resistance (7–9). Thus, there is an urgent need for a safe and effective therapeutic strategy to combat MRSA infections while delaying the development of drug resistance. One promising approach involves the use of antibiotic adjuvants or potentiators. Although these compounds typically exhibit little or no antimicrobial activity, they can enhance the efficacy of antibiotics by directly inhibiting bacterial resistance mechanisms, indirectly influencing other interdependent factors that contribute to resistance, and modulating host defense mechanisms (10, 11).

Natural compounds serve as a valuable source of potential antibiotic adjuvants, owing to their structural diversity and multi-target properties (12). Research has demonstrated that rare ginsenosides can enhance the sensitivity of drug-resistant bacteria to antibiotics (13). For example, the triterpenoid ginsenoside Rg3 has recently been proven to increase the sensitivity of MRSA to *β*-lactam and aminoglycoside antibiotics (14). Ginsenoside Rh2 (G-Rh2), another rare triplet ginsenoside, exhibits various pharmacological activities, including immunomodulation, anti-aging, and antitumor effects (15). Furthermore, G-Rh2 can significantly enhance the sensitivity of *S. aureus* strains to ciprofloxacin and levofloxacin (13). It also shows synergistic antibacterial effects against *Enterococcus faecalis* when combined with Ca(OH)_2_ (16). However, the antibacterial activities of G-Rh2 and *β*-lactam antibiotics against MRSA, along with the underlying molecular mechanisms, remain inadequately understood.

In this study, we conducted a comprehensive evaluation of the antibacterial effect of G-Rh2 in combination with *β*-lactam antibiotics against MRSA, as well as the potential underlying mechanisms. These findings may provide new insights into strategies for combating MRSA drug resistance.

## MATERIALS AND METHODS

### Bacterial strains and reagents

*S. aureus* ATCC 43300 (a standard MRSA strain) was obtained from the American Type Culture Collection (Manassas, VA, USA). Two clinical MRSA isolates, designated SA1 and SA2, were provided by the Microbiology Laboratory of Changshu Medical Laboratory. Bacteria were cultured on Mueller–Hinton agar (MHA) plates and suspended in Luria–Bertani broth (LB) or cation-adjusted Mueller–Hinton broth (CAMHB). MHA, LB, and CAMHB were purchased from Haibo Biotechnology (Qingdao, China). G-Rh2, cefoxitin (CFX), cefazolin (CFZ), ampicillin (AMP), meropenem (MEM), and vancomycin (VAN) were purchased from Chenguang Biotech Group Co., Ltd.

### Susceptibility testing and synergy testing

Susceptibility testing was conducted in accordance with Clinical and Laboratory Standards Institute (CLSI) guidelines (17). Minimum inhibitory concentrations (MICs) were determined using standardized broth microdilution. Specifically, twofold serial dilutions of G-Rh2 and other *β*-lactam antibiotics were prepared in CAMHB. Subsequently, 100 μL of each dilution was added to sterile 96-well plates, with a drug-free control group included. The concentration of the MRSA (*S. aureus* ATCC 43300, SA1, and SA2) bacterial suspension was adjusted to 1.0 × 10^6^ CFUs/mL during the logarithmic growth phase. Following this, 100 μL of the bacterial suspension was added to each well, and the plates were then cultured at 37°C for 16–20 h. The MIC was defined as the lowest drug concentration that exhibited no visible growth, and optical density (OD) at 600 nm was measured using a microplate reader (Thermo Fisher Scientific, Waltham, USA). The combined antibacterial effects were evaluated through the fractional inhibitory concentration index (FICI) derived from checkerboard assays. Briefly, 50 µL of continuously double-diluted G-Rh2 and antibiotics were added to the horizontal and vertical wells of a 96-well plate, respectively. Then, 100 μL of MRSA bacterial suspension (1.0 × 10^6^ CFUs/mL) was added to each well, and the plate was cultured at 37°C for 16–20 h. The MIC of G-Rh2 and antibiotics in combination against MRSA was recorded, and OD 600 nm of each well was measured. Each experiment was performed with at least three biological replicates. The FICI was calculated as follows: FICI = (MIC of G-Rh2 in combination/MIC of G-Rh2 alone) + (MIC of antibiotic in combination/MIC of antibiotic alone). The FICI interpretations were as follows: ≤0.5, synergy; 0.5–1, partial synergy; 1–4, additive or no effect; >4, antagonism. The antibacterial rate was calculated as follows: Antibacterial Rate = (OD 600 nm of the experimental group/OD 600 nm of the control group) × 100%.

### Growth curve of bacteria

*S. aureus* ATCC 43300 was cultured to the logarithmic growth phase and subsequently diluted to 1.0 × 10^6^ CFU/mL. Bacterial suspensions were incubated with various concentrations of G-Rh2 and four *β*-lactam antibiotics, either alone or in combination, for 24 h at 37°C while shaking at 180 rpm. A control group without treatment was included for comparison. Optical density at 600 nm was measured for each sample at 0, 2, 4, 6, 8, 10, 12, and 24 h. For cultures treated with G-Rh2, CFX, the G-Rh2/CFX combination, and the control group, samples were collected at 12 h, and serial 10-fold dilutions were prepared to determine bacterial density through plate counting. The experiment was repeated in triplicate.

### Resistance induction assay

The resistance induction assay of *S. aureus* ATCC 43300 to CFX and VAN was adapted from Zhang et al. (18). Briefly, a culture of *S. aureus* ATCC 43300 (5.0 × 10^5^ CFUs/mL) was added to each well of a 96-well plate containing a serially diluted concentration gradient of CFX and VAN, either alone or in combination with G-Rh2 (6.25 μg/mL). After incubation at 37°C for 16 h, the MICs of the antibiotic in each group were recorded. Subsequently, 100 µL of culture from each group at a concentration of 1/2 MIC antibiotic was removed and added to 5 mL of LB, followed by incubation at 37°C for 6 h. The MICs for the second-generation bacteria were then determined, and these procedures were repeated for 16–20 generations.

### Observation of bacterial morphology

*S. aureus* ATCC 43300 was cultivated to the logarithmic growth phase until the OD 600 nm reached 0.6. The bacterial suspensions were incubated at 37°C for 6 h with G-Rh2 (2 μg/mL), CFX (12.5 μg/mL), or their combination, while an untreated negative control was also included. After incubation, cells were pelleted by centrifugation at 8,000 rpm for 5 min at 4°C and subsequently fixed in 2.5% glutaraldehyde for 12 h. The samples were then washed three times with phosphate-buffered saline (PBS) and dehydrated using an ethanol gradient (30%, 50%, 70%, 90%, 100%). Finally, the specimens were dried, sputter-coated with gold, and examined using scanning electron microscopy (SEM) (JEOL, Tokyo, Japan).

### Leakage of proteins and nucleic acids

An overnight culture of *S. aureus* ATCC 43300 was washed and resuspended in PBS. Bacterial suspensions at a concentration of 5.0 × 10^7^ CFUs/mL were incubated with G-Rh2 (2 μg/mL), CFX (12.5 μg/mL), or their combination at 37°C for 4 h, while an untreated suspension served as the negative control. After incubation, samples were centrifuged at 13,000 rpm for 10 min, and the supernatants were collected. The absorbance of the supernatants was measured at 260 nm (nucleic acids) and 280 nm (proteins) using a spectrophotometer (Bio-Tek Instruments Inc., Winooski, VT, USA) (19).

### Biofilm inhibition assays

*S. aureus* ATCC 43300 was cultured to the logarithmic growth phase, and the inoculum was adjusted to 1.0 × 10^7^ CFUs/mL. Bacterial suspensions mixed with G-Rh2, CFX, or the G-Rh2/CFX combinations were seeded into 96-well plates and incubated at 37°C for 24 h. A control group without drug treatment was included for comparison. After incubation, planktonic cells were removed, and each well was gently washed twice with 200 µL of PBS to remove remaining free-floating bacteria. The wells were air-dried and stained with 200 µL of 0.1% crystal violet for 30 min. The dye was removed, wells were washed twice with PBS and dried, and biofilms were destained using 200 µL of 95% ethanol for 15 min. The biofilm was quantified by measuring its absorbance at a wavelength of 570 nm.

### Biofilm eradication assays

The overnight-cultured *S. aureus* ATCC 43300 bacterial suspension was adjusted to a concentration of 1.0 × 10^7^ CFUs/mL. This suspension was then seeded into 96-well plates and incubated for 24 h to facilitate mature biofilm formation. After the planktonic cells were removed, the wells were washed twice with 200 µL of PBS to eliminate remaining non-adherent bacteria. The indicated concentrations of G-Rh2, CFX, or the G-Rh2/CFX combinations were then added, and the plates were incubated at 37°C for 24 h. The control group that remained untreated with any drug was included. Biofilm quantification was performed using crystal violet staining as previously described.

### Assessment of bacterial viability within mature biofilms

*S. aureus* ATCC 43300 was cultured until it reached the logarithmic growth phase, after which the bacterial suspension was adjusted to 1.0 × 10^7^ CFUs/mL. Coverslips were placed in a 6-well plate, and 2 mL of the bacterial suspension was added to each well. The plate was then incubated at 37°C for 24 h to promote mature biofilm formation. Following this incubation, the culture was removed, and each well was rinsed with PBS. The wells were subsequently incubated for 24 h with G-Rh2 (2 μg/mL), CFX (12.5 μg/mL), or their combination. The negative control group received unmedicated LB treatment. Afterward, the cultures were removed, and the cover slips from each group were washed three times with PBS to eliminate surface airborne bacteria. Following the manufacturer’s instructions for the bacterial viability detection kit, the samples were stained with a mixture of SYTO-9 and propidium iodide (PI) for 20 min. Live and dead bacteria within the mature biofilms were visualized using confocal laser scanning microscopy (CLSM) (Olympus, Tokyo, Japan).

### Transcriptomic analysis

The overnight-cultured *S. aureus* ATCC 43300 bacterial suspension was adjusted to a concentration of 1.0 × 10^8^ CFUs/mL. The bacterial suspension was then exposed to G-Rh2 (12.5 µg/mL), CFX (0.5 µg/mL, 1/16 MIC), or their combination at 37°C for 6 h. The sample solvent served as the negative control in this experiment. Total RNA was extracted from each sample using the TRIzol Reagent/RNeasy Mini Kit (Qiagen) and subsequently quantified and assessed for quality. Library construction and Illumina sequencing were conducted by Azenta China. All RNA-seq reads were aligned to the genome of *S. aureus* ATCC 43300 (https://www.ncbi.nlm.nih.gov/datasets/genome/GCF_046741765.1/; accessed on 19 January 2025) for downstream analysis. Pairwise comparisons of gene expression between groups were performed, and genes exhibiting an adjusted *P* value < 0.05 and |fold change| > 2 were classified as differentially expressed genes (DEGs). To interpret the biological functions and pathways associated with the identified DEGs, functional enrichment analysis was conducted. Gene Ontology (GO) enrichment analysis and Kyoto Encyclopedia of Genes and Genomes (KEGG) pathway analysis were performed. Terms and pathways with an adjusted *P* value < 0.05 were considered significantly enriched.

### Quantitative reverse transcription PCR

To validate the transcriptomic results, 10 DEGs were selected for quantitative reverse transcription PCR (qRT-PCR) analysis. In alignment with the transcriptome experiment, *S. aureus* ATCC 43300 was treated with G-Rh2, CFX, and their combination, while a negative control remained untreated. Total RNA was extracted from the experimental bacterial strain using the Bacteria RNA Enhancement Reagent (Vazyme, Nanjing, China) and the FastPure Complex Tissue/Cell Total RNA Isolation Kit (Vazyme, Nanjing, China). Reverse transcription to synthesize complementary DNA was performed using HiScript III RT SuperMix for qPCR (+gDNA wiper) (Vazyme, Nanjing, China). The primers (Sangon Biotech, Shanghai, China) utilized for the qRT-PCR reactions are shown in Table S1. qRT-PCR was performed on an Applied Biosystems QuantStudio 5 PCR instrument (Thermo Fisher Scientific, Waltham, USA) with ChamQ Blue Universal SYBR qPCR Master Mix (Vazyme, Nanjing, China). The *16S rRNA* gene served as the reference gene, and the 2^(−ΔΔCT)^ method was employed to quantify the relative mRNA expression.

### Statistical analysis

Statistical analysis involved conducting one-way ANOVA, followed by Duncan’s test using SPSS Statistics (version 27, IBM Company, Chicago, IL, USA). Prism (version 10, Dotmatics, Bishop’s Stortford, UK) was utilized for data visualization. A *P* value < 0.05 denoted statistical significance.

## RESULTS

### Synergistic antibacterial activity of G-Rh2 with *β*-lactams against MRSA

**Fig 1:**
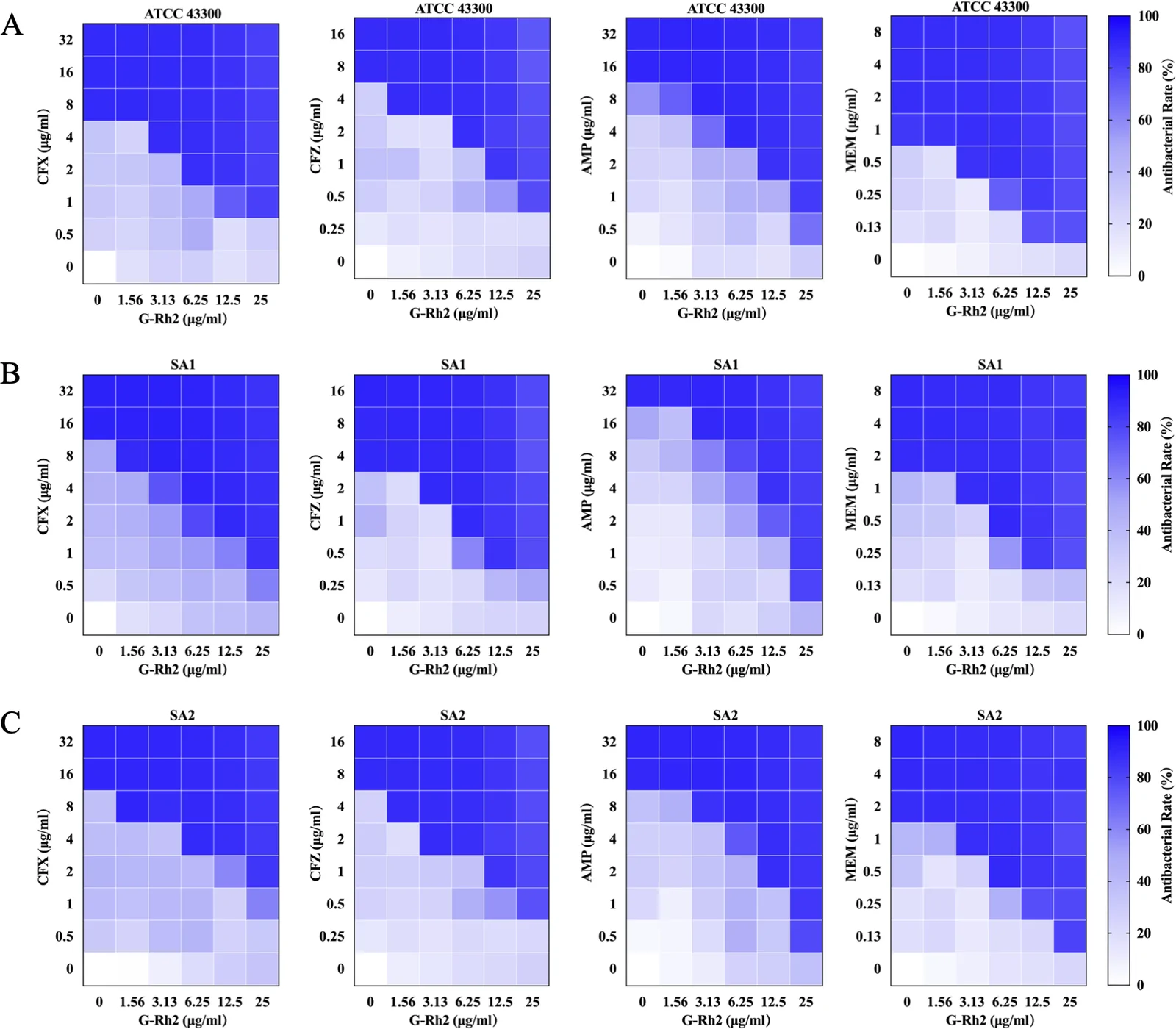
The antibacterial activities of varying concentration combinations of G-Rh2 and *β*-lactam antibiotics (CFX, CFZ, AMP, and MEM) against MRSA strains. (**A**) Antibacterial rates against *S. aureus* ATCC 43300; (**B**) antibacterial rates against *S. aureus* SA1; (**C**) antibacterial rates against *S. aureus* SA2. Darker blue shading indicates stronger antibacterial activity.

According to CLSI guidelines, all *S. aureus* strains in this study meet the criteria for MRSA, defined as an MIC of CFX ≥ 8 µg/mL. The MIC ranges for the four tested *β*-lactam antibiotics (CFX, CFZ, AMP, and MEM) varied from 1 to 32 μg/mL among the MRSA strains (Table 1). The maximum soluble concentration of G-Rh2 tested was 100 μg/mL. At this concentration, G-Rh2 did not achieve complete inhibition of visible growth of any MRSA strain after 16–20 h of incubation, although a slight reduction in turbidity was observed. Therefore, the MICs of G-Rh2 against all tested MRSA strains were consistently recorded as >100 µg/mL. We evaluated the combined antibacterial effect of G-Rh2 with four *β*-lactam antibiotics using a checkerboard assay. When the concentration of added G-Rh2 reached 6.25 μg/mL, the MICs of the four *β*-lactam antibiotics against three MRSA strains decreased by at least fourfold. The FICI values were all <0.5, indicating a synergistic interaction between G-Rh2 and the *β*-lactam antibiotics (Table 1). Importantly, this synergistic effect was consistently observed not only in the reference strain ATCC 43300 but also in the two clinical MRSA isolates SA1 and SA2, confirming the clinical relevance of the combination. When combined with 6.25 μg/mL G-Rh2, the MIC of *S. aureus* ATCC 43300 to CFX decreased to 2 μg/mL. According to CLSI guidelines (17), when the MIC of CFX is ≤4 µg/mL, *S. aureus* is considered sensitive to methicillin. Therefore, the experimental results demonstrate that G-Rh2 can resensitize MRSA to *β*-lactam antibiotics. Furthermore, G-Rh2 enhanced the antibacterial activity of the *β*-lactam antibiotics against MRSA in a dose-dependent manner (Fig. 1A through C).

**TABLE 1:** Antimicrobial activity of G-Rh2 and *β*-lactam antibiotics against MRSA, alone and in combination^*a*^

MRSA isolates	Compounds	MICa (µg/mL)	MICc (µg/mL)	FICI	Interactions
ATCC 43300	G-Rh2	>100	6.25	0.3125	Synergism
	CFX	8	2		
	G-Rh2	>100	6.25	0.3125	Synergism
	CFZ	8	2		
	G-Rh2	>100	6.25	0.3125	Synergism
	AMP	16	4		
	G-Rh2	>100	6.25	0.3125	Synergism
	MEM	1	0.25		
SA1	G-Rh2	>100	6.25	0.1875	Synergism
	CFX	16	2		
	G-Rh2	>100	6.25	0.3125	Synergism
	CFZ	4	1		
	G-Rh2	>100	6.25	0.3125	Synergism
	AMP	32	8		
	G-Rh2	>100	6.25	0.3125	Synergism
	MEM	2	0.5		
SA2	G-Rh2	>100	6.25	0.3125	Synergism
	CFX	16	4		
	G-Rh2	>100	6.25	0.3125	Synergism
	CFZ	8	2		
	G-Rh2	>100	6.25	0.3125	Synergism
	AMP	16	4		
	G-Rh2	>100	6.25	0.3125	Synergism
	MEM	2	0.5		

^*a*^ MICa, minimum inhibitory concentration of a single compound; MICc, minimum inhibitory concentration of compounds in combination.

### Time-dependent bactericidal effect of the combination

**Fig 2:**
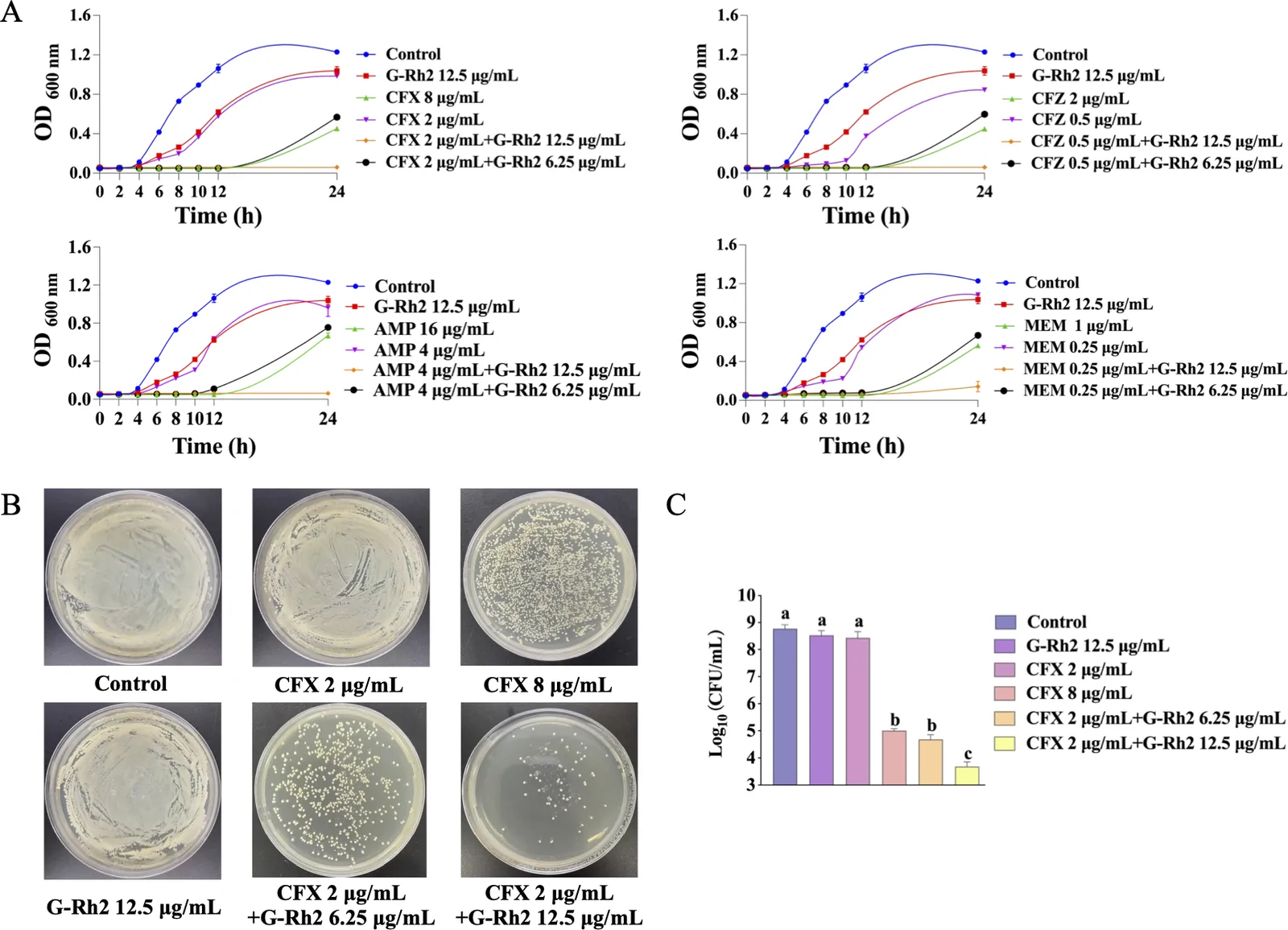
Effects of G-Rh2 and *β*-lactam antibiotics, administered alone or in combination, on the growth curves of *S. aureus* ATCC 43300. (**A**) Growth curves were recorded following the application of G-Rh2 and CFX, CFZ, AMP, and MEM to *S. aureus* ATCC 43300. Data are presented as means ± SD from three independent experiments. (**B**) Representative photographs of agar plates from one of three independent experiments following 10-fold serial dilution of the treated bacterial suspensions at 12 h. (**C**) Plate-count statistics for *S. aureus* ATCC 43300 treated with G-Rh2 and CFX, either alone or in combination, over a 12-h period. Data represent the averages of three independent experiments (mean ± SD). Different letters above the bars indicate statistically significant differences between treatments (*P* < 0.05), while identical letters denote no significant difference (*P* > 0.05).

Based on the observed synergistic antimicrobial effect of G-Rh2 in combination with four *β*-lactam antibiotics against *S. aureus* ATCC 43300, we examined the effects of G-Rh2 and *β*-lactam antibiotics on bacterial growth curves. As shown in Fig. 2A, sub-MIC *β*-lactam antibiotics (1/4 MIC CFX, 1/4 MIC CFZ, 1/4 MIC AMP, and 1/4 MIC MEM) and 12.5 μg/mL G-Rh2 alone only slightly reduced exponential-phase growth of *S. aureus* ATCC 43300. In contrast, the addition of 6.25 μg/mL G-Rh2 to sub-MIC *β*-lactam antibiotics enhanced growth inhibition. At a G-Rh2 concentration of 12.5 μg/mL, all combined treatment groups effectively suppressed bacterial growth for 24 h. Consistently, plate-count assays at 12 h showed that the combination of CFX and G-Rh2 significantly reduced CFU compared with both the control and the respective single-agent groups, indicating a synergistic bactericidal effect. Among all treatment groups, the combination of 1/4 MIC CFX with 12.5 μg/mL G-Rh2 produced the most pronounced bactericidal activity (Fig. 2B and C).

### G-Rh2 delays the development of antibiotic resistance

**Fig 3:**
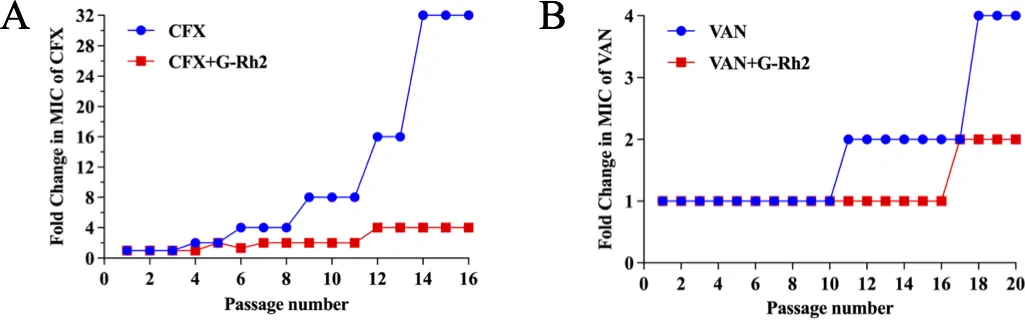
Development of resistance in *S. aureus* ATCC 43300 during serial passaging in the presence of sub-MIC antimicrobials, with or without G-Rh2. (**A**) The resistance development of *S. aureus* ATCC 43300 to CFX was assessed with or without 6.25 μg/mL G-Rh2. (**B**) The resistance development of *S. aureus* ATCC 43300 to VAN was evaluated with or without 6.25 μg/mL G-Rh2.

Several previous studies have demonstrated that bacteria can acquire resistance when exposed for prolonged periods to antibiotic concentrations below the MIC (20, 21). We investigated the effects of sub-MIC antibiotics combined with or without G-Rh2 on the induced resistance of *S. aureus* ATCC 43300. When stimulated by sub-MIC levels of CFX, *S. aureus* ATCC 43300 exhibited an increase in MIC by the fourth generation of culture, followed by a continuous rise in MIC. In contrast, the presence of G-Rh2 resulted in a slower increase in the MIC of CFX (Fig. 3A). Similarly, exposure to sub-MIC levels of VAN led to an increase in MIC by the tenth generation of culture; however, the combination of VAN and G-Rh2 mitigated the development of resistance to VAN (Fig. 3B).

### Morphological changes of the cell envelope

**Fig 4:**
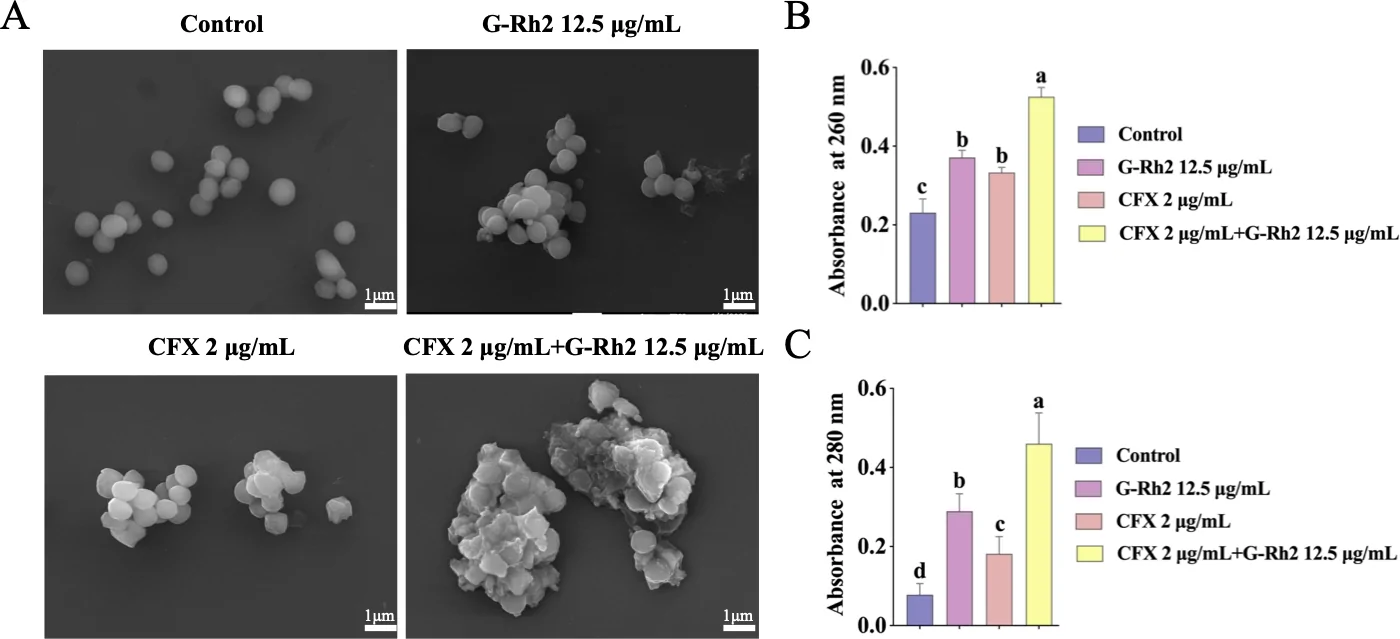
Effects of combined G-Rh2 and CFX on cell membrane integrity in *S. aureus* ATCC 43300. (**A**) SEM images of cells after treatment with G-Rh2 and CFX, alone or in combination. Scale bar: 1 μm. (**B**) Release of nucleic acids, measured as absorbance at 260 nm, following treatment with G-Rh2 and CFX, alone or in combination. (**C**) Release of proteins, measured as absorbance at 280 nm, following treatment with G-Rh2 and CFX, alone or in combination. Data represent the mean ± SD of three independent experiments. Different letters above the bars indicate statistically significant differences between treatments (*P* < 0.05), while identical letters denote no significant difference (*P* > 0.05).

SEM is widely used to evaluate the effect of antimicrobial agents on the bacterial cell envelope (22). We therefore employed SEM to examine the morphological changes of *S. aureus* ATCC 43300 following treatments with G-Rh2, CFX, or their combination. As shown in Fig. 4A, the *S. aureus* cells in the control group appeared round and smooth. In the G-Rh2-treated group, the cells largely retained their normal shape, but a small number exhibited swelling and rupture. The bacterial morphology in the CFX-treated group remained largely unchanged; however, the surfaces of some bacteria displayed signs of shrinkage and collapse. In contrast, the combined treatment group revealed a roughened bacterial surface, accompanied by significant contraction, deformation, swelling, or rupture, indicating severe disruption of the cell envelope.

### Leakage of intracellular contents

Loss of cell membrane integrity leads to the leakage of intracellular components, such as nucleic acids and proteins, which can be quantified by measuring the absorbance of the supernatant at 260 and 280 nm, respectively. As shown in Fig. 4B and C, treatment of *S. aureus* ATCC 43300 with G-Rh2 or CFX alone caused a moderate increase in nucleic acid and protein release compared with the untreated control, whereas their combination resulted in a substantially greater leakage. Specifically, the combined treatment group exhibited 1.42- and 1.58-fold higher nucleic acid release than the G-Rh2-treated and CFX-treated groups, respectively, and 1.59- and 2.53-fold higher protein release. These results indicate that the combination of G-Rh2 and CFX acts synergistically to compromise cell membrane integrity.

### G-Rh2 and CFX synergistically inhibit biofilm formation

**Fig 5:**
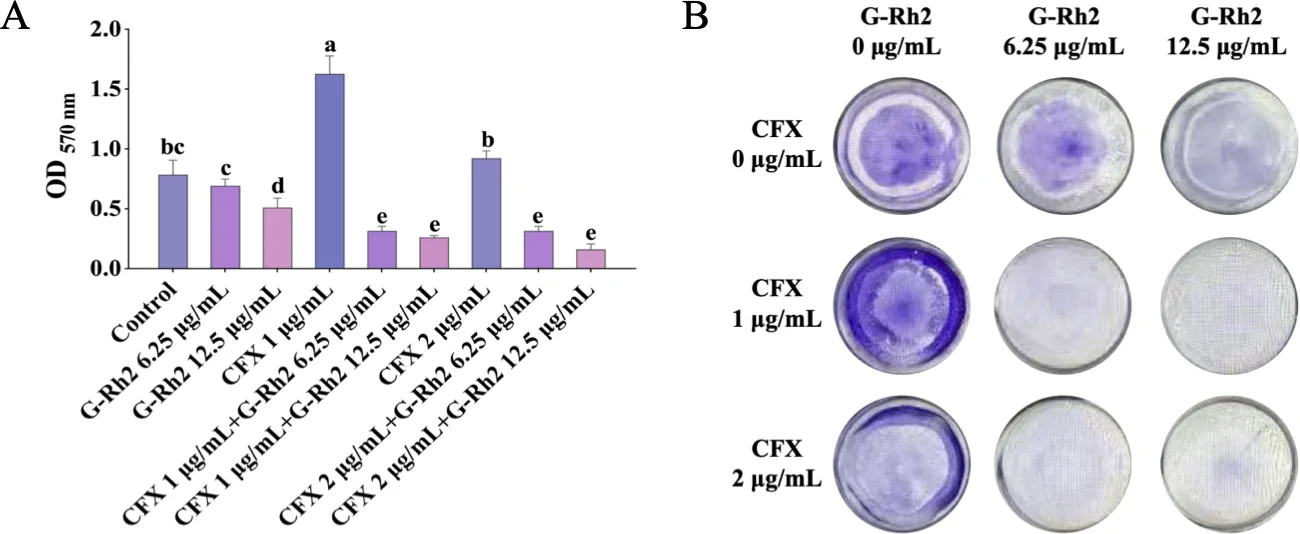
Effects of combined G-Rh2 and CFX on biofilm formation in *S. aureus* ATCC 43300. (**A**) The effects of G-Rh2 and CFX, alone and in combination, on biofilm formation were evaluated using crystal violet staining. Data represent the mean ± SD of three independent experiments. Different letters above the bars indicate statistically significant differences between treatments (*P* < 0.05), while identical letters denote no significant difference (*P* > 0.05). (**B**) Representative image of crystal violet-stained biofilms.

Bacterial biofilm formation plays a significant role in antimicrobial resistance. To evaluate the effect of G-Rh2 on biofilm formation in the presence of a *β*-lactam, we selected CFX as a representative and treated *S. aureus* ATCC 43300 with G-Rh2, CFX, or their combinations. Biofilm formation was assessed using the crystal violet staining method. As illustrated in Fig. 5A, G-Rh2 at a concentration of 12.5 μg/mL effectively inhibited biofilm formation. In contrast, sub-MIC concentrations of CFX did not inhibit biofilm formation and, at 1/8 MIC, even promoted it. However, when CFX was combined with G-Rh2, a synergistic effect was observed, resulting in a more pronounced inhibition of biofilm formation (Fig. 5A and B).

### G-Rh2 and CFX synergistically eradicate mature biofilms

**Fig 6:**
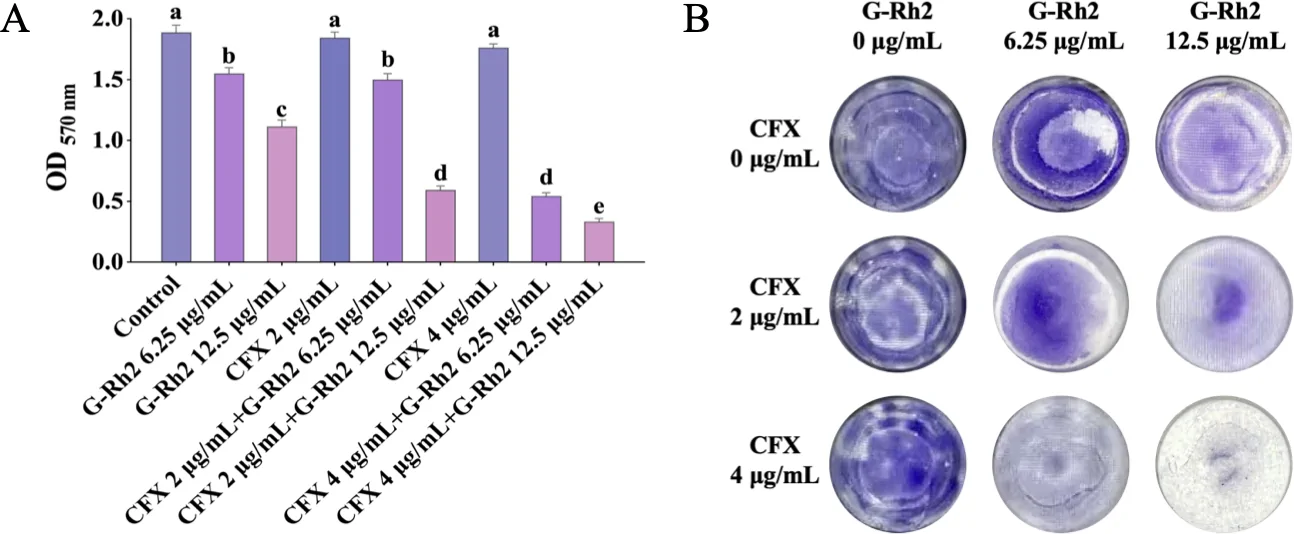
Effects of combined G-Rh2 and CFX on mature biofilms formed by *S. aureus* ATCC 43300. (**A**) The effects of G-Rh2 and CFX, alone and in combination, on the eradication of mature biofilms were evaluated by crystal violet staining. Data represent the mean ± SD of three independent experiments. Different letters above the bars indicate statistically significant differences between treatments (*P* < 0.05), while identical letters denote no significant difference (*P* > 0.05). (**B**) Representative image of crystal violet-stained biofilms.

**Fig 7:**
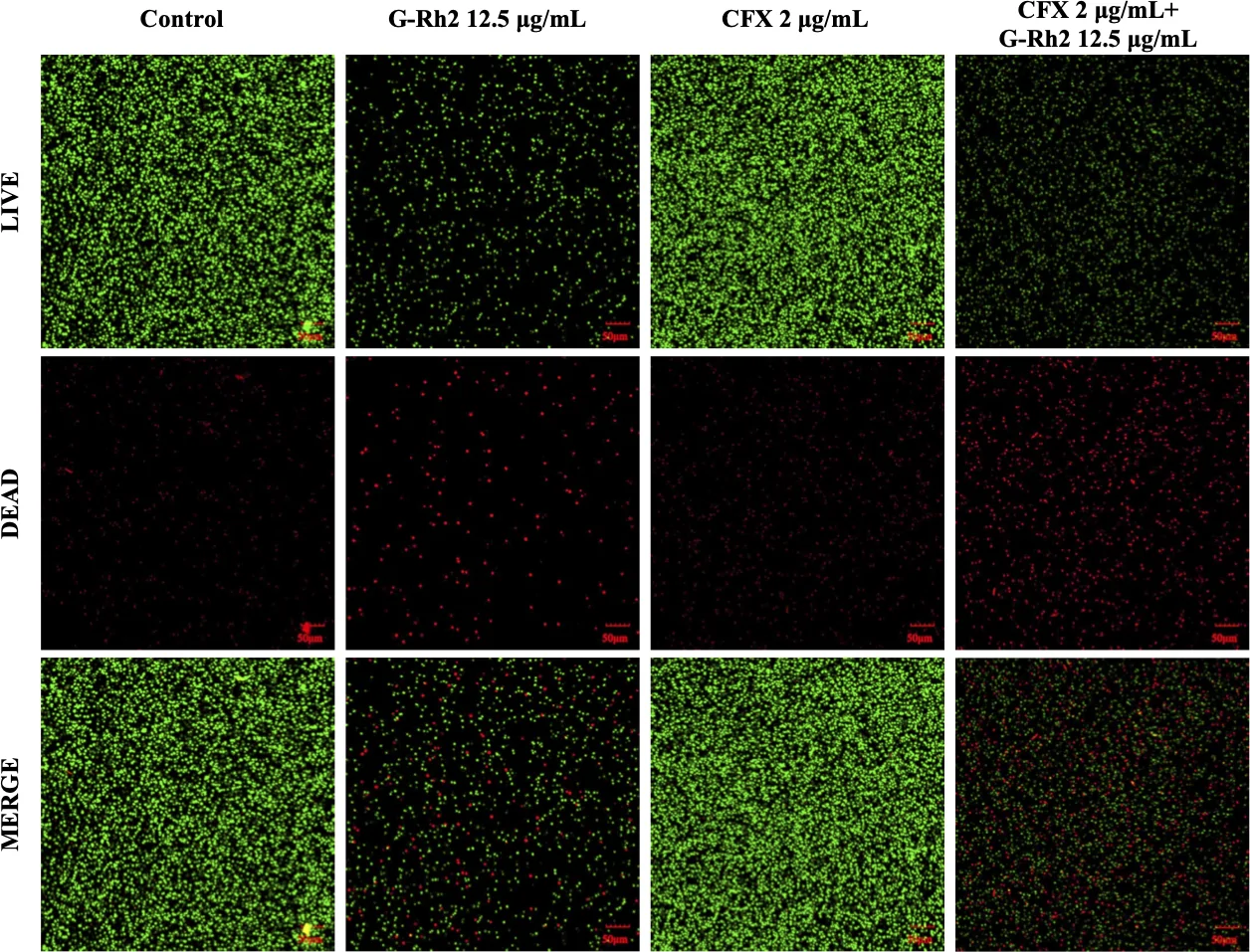
CLSM photographs of live and dead bacteria in mature biofilms after treatment with G-Rh2 (12.5 μg/mL) and CFX (2 μg/mL) alone or in combination. Scale bar: 50 µm.

We next investigated whether the combination could eradicate preformed mature biofilms. Using CFX as the representative *β*-lactam, mature biofilms of *S. aureus* ATCC 43300 were treated with G-Rh2, CFX, or their combinations and analyzed by crystal violet staining. As shown in Fig. 6A and B, G-Rh2 alone demonstrated effective eradication of mature biofilms at concentrations exceeding 6.25 μg/mL. In contrast, sub-MIC CFX alone failed to achieve significant eradication. However, the addition of G-Rh2 to sub-MIC CFX resulted in a synergistic interaction, leading to markedly enhanced biofilm clearance (Fig. 6A and B). We further evaluated the effect of the combination of G-Rh2 and CFX on mature biofilms through CLSM. SYTO 9 is a green, fluorescent nucleic acid dye that penetrates intact cell membranes, staining both live and dead bacteria. PI is a red fluorescent nucleic acid dye that selectively stains bacteria with compromised cell membranes. As shown in Fig. 7, both the control and CFX-treated groups exhibited dense green fluorescence over large areas with minimal red fluorescence, indicating intact biofilm structures consisting predominantly of viable bacteria. In contrast, the G-Rh2-treated group showed a looser biofilm architecture along with sporadic red signals. This suggests that G-Rh2 can disrupt mature biofilms, though its antibacterial activity appears limited. Furthermore, the combination treatment group displayed not only a dispersed biofilm structure but also a marked increase in red fluorescence within the biofilm. These results demonstrate that the combination of G-Rh2 and CFX effectively eradicates mature biofilms and kills the embedded bacteria.

### Transcriptomic analysis reveals downregulation of two-component system and ATP-binding cassette transporter pathways

**Fig 8:**
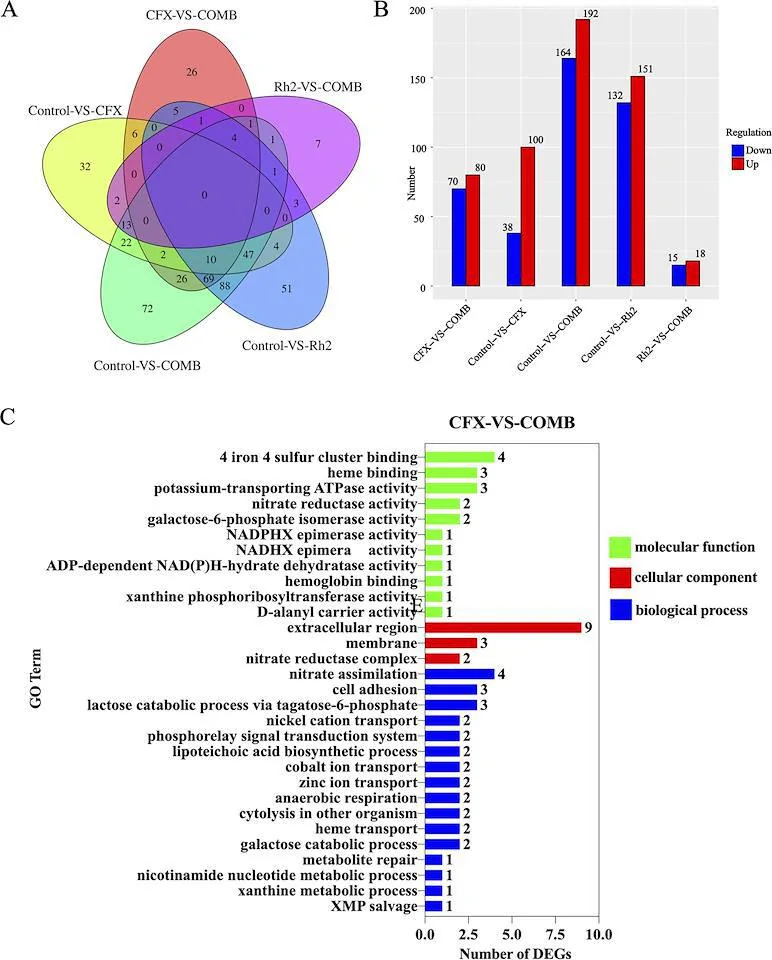
Changes in the transcriptome of *S. aureus* ATCC 43300 treated with G-Rh2, CFX, their combination, or a vehicle control. (**A**) Venn diagram showing the number of DEGs in pairwise comparisons among treatment groups and the number of shared DEGs. (**B**) A comparison of the numbers of up-regulated and down-regulated DEGs across the treatment groups. (**C**) GO distribution of DEGs between the CFX-treated group and the combined treatment group.

**Fig 9:**
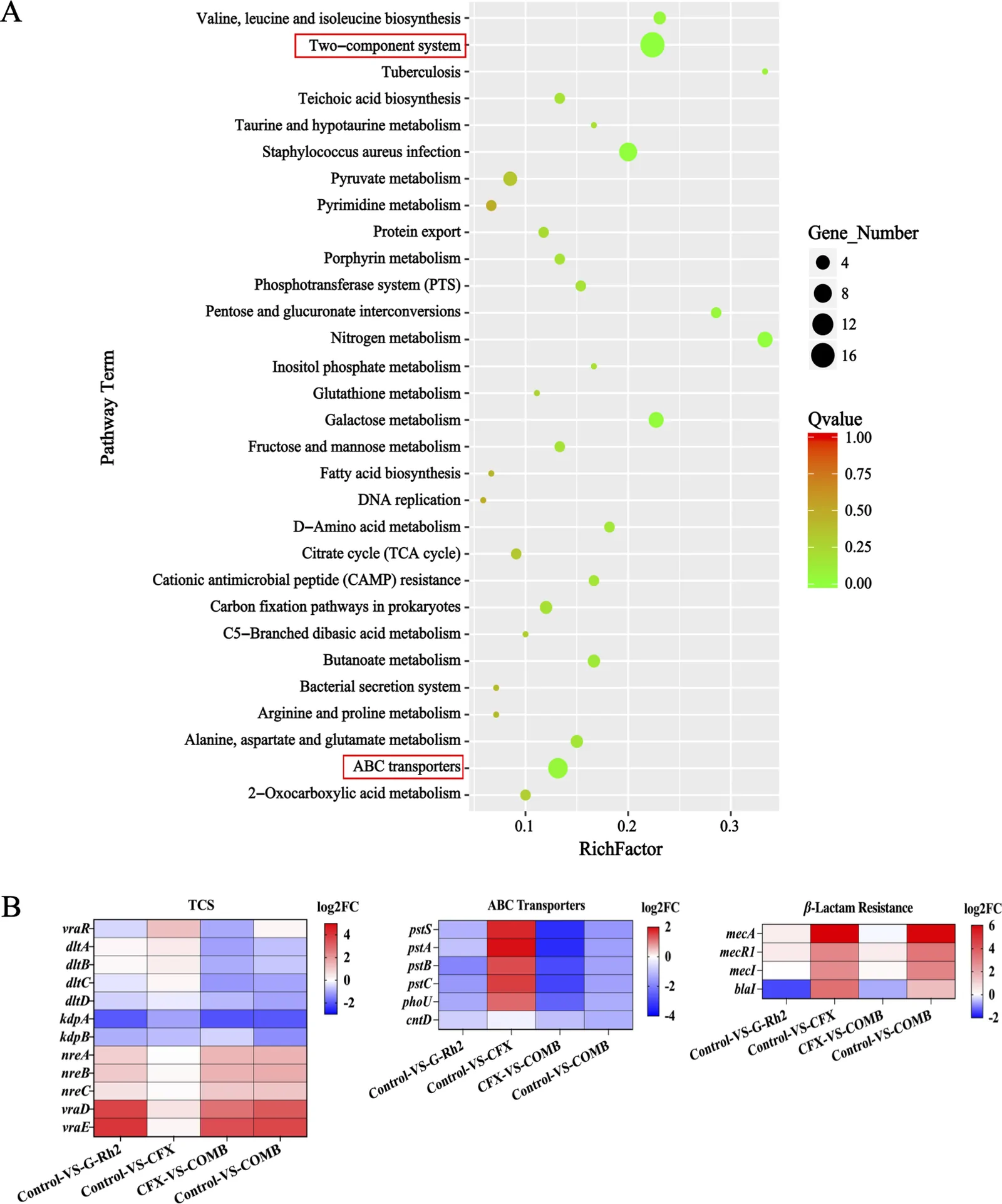
KEGG pathway enrichment analysis. (**A**) KEGG enrichment scatter plots for DEGs resulting from the CFX-treated group and the combined treatment group. (**B**) Heatmap of selected DEGs involved in the TCS, ABC transporters, and *β*-lactam resistance.

**Fig 10:**
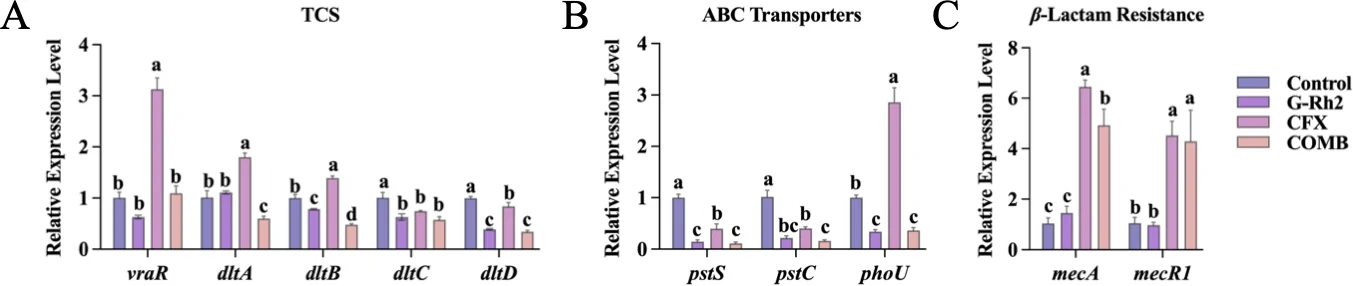
Validation of transcriptome results for selected genes by RT-qPCR. (**A**) Relative expression of genes involved in TCS. (**B**) Relative expression of genes involved in ABC transporters. (**C**) Relative expression of genes involved in *β*-lactam resistance. Results are shown as fold changes relative to the control (set to 1). Data represent three biological replicates (mean ± SD). Different letters above the bars indicate statistically significant differences between treatments (*P* < 0.05); identical letters indicate no significant difference (*P* > 0.05).

To elucidate the molecular mechanism underlying the synergistic antibacterial effect of G-Rh2 and *β*-lactam antibiotics, we performed transcriptome sequencing on *S. aureus* ATCC 43300 treated with G-Rh2, CFX, their combination, or a vehicle control. The transcriptome analysis identified a total of 493 DEGs across all comparisons (Fig. 8A). Relative to the control group, the CFX-treated, G-Rh2-treated, and combined treatment groups showed 100, 151, and 192 upregulated genes, along with 38, 132, and 164 downregulated genes, respectively (Fig. 8B). In comparison to the CFX-treated group, the combined treatment group displayed 80 upregulated genes and 70 downregulated genes (Fig. 8B). GO enrichment analysis was conducted to clarify the molecular functions (MF), cellular components (CC), and biological processes (BP) associated with the DEGs. As shown in Fig. 8C, a comparison between the combined treatment group and the CFX-treated group revealed a total of 11 terms related to MF, 3 terms for CC, and 16 terms for BP. Specifically, DEGs in the CC category were predominantly associated with the extracellular region and the plasma membrane. Within the BP category, salient enriched terms included nitrate assimilation and cell adhesion. This further suggests that G-Rh2 may influence the structure of cell membranes and diminish cell adhesion. KEGG pathway enrichment analysis revealed predominant enrichment of DEGs in the two-component system (TCS) and ATP-binding cassette (ABC) transporter pathways. This was observed in the comparisons between the combined treatment and CFX-treated groups, and between the G-Rh2-treated and control groups (Fig. 9A; Fig. S1 and S3). In the comparisons of the combined treatment or CFX-treated groups with the control group, DEGs were also enriched in the *β*-lactam resistance pathway, alongside the previously mentioned pathways (Fig. S2 and S3). In a further comparison of the combined treatment group and the CFX-treated group, the addition of G-Rh2 significantly reduced the expression of genes involved in TCS, such as *vraR*, *dltA*, *dltB*, *dltC*, *dltD*, *kdpA*, and *kdpB*, as well as genes associated with ABC transporters, including *pstS*, *pstA*, *pstB*, *pstC*, *phoU*, and *cntD*. Notably, G-Rh2 did not influence the expression of *β*-lactam resistance genes (*mecA*, *mecR1*, and *mecI*) (Fig. 9B). To validate the transcriptome data, we performed qRT-PCR on 10 key DEGs from the above pathways: *pstS*, *pstC*, *phoU*, *vraR*, *dltA*, *dltB*, *dltC*, *dltD*, *mecA*, and *mecR1*. The gene expression trends observed in the qRT-PCR analysis were consistent with the transcriptome results, thereby confirming the accuracy of the transcriptomic analysis (Fig. 10).

## DISCUSSION

MRSA exhibits resistance to nearly all *β*-lactam drugs, except fifth-generation cephalosporins, due to the presence of the PBP2a protein (23). In a 2024 report by the World Health Organization, MRSA was identified as one of 15 bacterial priority pathogens categorized as high priority. MRSA infections remain widespread globally, presenting significant public health challenges. Consequently, innovative treatment strategies and the development of novel antimicrobials are urgently required to combat the issue of antimicrobial resistance among priority pathogens (24). Natural compounds represent a crucial source of novel antibacterial agents. Numerous natural compounds have demonstrated efficacy as antibacterial adjuvants, particularly in enhancing the effectiveness of conventional antibiotics against MRSA infections. For instance, the combination of piperine and gentamicin displays significant antibacterial activity against MRSA (25). Ginsenosides are the primary active constituents of ginseng, American ginseng, and Notoginseng. Studies indicate that ginsenosides with lower polarity, such as Rh2, Rk3, Rh4, and Rg5, show great potential for antibacterial effects against bacteria and fungi (26, 27). G-Rh2 has been shown to increase intracellular accumulation of ciprofloxacin and levofloxacin in *S. aureus* by inhibiting efflux transporters, thereby producing a synergistic antibacterial effect (28, 29). In this study, we demonstrate that G-Rh2 can resensitize MRSA to *β*-lactam antibiotics and exhibits synergistic anti-biofilm activity in conjunction with these antibiotics.

Using checkerboard assays, we assessed the antibacterial effects of G-Rh2 in combination with four *β*-lactam antibiotics (CFX, CFZ, AMP, and MEM) against a standard MRSA strain and two clinical MRSA isolates. The results indicated a synergistic antibacterial effect between G-Rh2 and the four *β*-lactam antibiotics. Notably, a concentration of 6.25 µg/mL of G-Rh2 effectively restored MRSA sensitivity to *β*-lactam antibiotics. Additionally, the antibacterial sensitizing effect of G-Rh2 exhibited a dose-dependent relationship. Bacterial growth curve assays indicated that sub-MIC *β*-lactam antibiotics, when combined with G-Rh2 at 12.5 µg/mL, consistently inhibited MRSA growth. Moreover, G-Rh2 markedly delays the development of MRSA resistance induced by low concentrations of CFX or VAN. These results indicate that the combination of G-Rh2 with *β*-lactam antibiotics may offer novel strategies for addressing MRSA drug resistance.

The bacterial cell envelope is a complex, multilayered structure that is central to *S. aureus* pathogenesis and serves as the primary target for many clinically used antibiotics. In Gram-positive bacteria, such as *S. aureus*, this envelope is composed of the cytoplasmic membrane and a cell wall, which together maintain cell shape and protect against environmental insults (30, 31). SEM observations revealed that the combination of G-Rh2 and CFX induced severe morphological alterations in MRSA, including surface roughening, contraction, deformation, and rupture, suggesting extensive disruption of the cell envelope. To further evaluate whether this structural disruption compromised cell membrane integrity, we measured the leakage of intracellular nucleic acids and proteins. The combination of G-Rh2 and CFX resulted in substantially greater leakage compared with either agent alone, indicating a loss of cell membrane integrity. Notably, G-Rh2 alone already caused a moderate increase in leakage despite its limited bactericidal activity, suggesting that G-Rh2 itself can compromise membrane integrity. Previous studies have shown that the cell membrane is a primary target for ginsenosides. Ginsenosides can alter membrane biophysical properties, such as fluidity and permeability, thereby exerting antibacterial effects (32). Korean red ginsenosides have been shown to enhance the antibacterial activity of kanamycin against MRSA. When a bacterial membrane mimic liposome containing a fluorescent marker is treated with ginsenosides, there is an accelerated leakage of fluorescent dyes, suggesting that ginsenosides can enhance membrane permeability (33). We therefore speculate that G-Rh2 may enhance the sensitivity of MRSA to β-lactam antibiotics, at least in part, by compromising cell membrane integrity.

Beyond planktonic bacteria, biofilms represent a significant challenge in the treatment of MRSA infections due to their role in enhancing drug resistance and fostering infection persistence (34). Bacteria establish biofilms by adhering to tissues and the surfaces of indwelling medical devices. This adhesion depends on surface hydrophobicity, the presence of fimbriae and flagella, and the production of extracellular polysaccharides (EPS). Studies have found that saponins can diminish surface hydrophobicity, thereby reducing bacterial adhesion (16, 35). G-Rh2, a saponin compound, has been previously demonstrated to disrupt biofilms formed by certain cariogenic bacteria by inhibiting bacterial activity and eliminating EPS (16, 36). In our study, sub-MIC CFX did not inhibit or eradicate biofilms formed by MRSA and even induced biofilm formation, consistent with previous findings (37). When administered alone, G-Rh2 inhibited biofilm development at concentrations exceeding 12.5 μg/mL and disrupted mature biofilms at concentrations above 6.25 μg/mL. Significantly, the combination of CFX and G-Rh2 exhibited synergistic anti-biofilm activity. The staining experiment of live and dead bacteria in the biofilm further proved that this combination can effectively eradicate mature biofilms and kill embedded bacteria. Feng et al. reported that propolis nanoparticles can decrease biofilm compactness to allow antibiotics to penetrate and exhibit significant synergistic anti-biofilm activity (38). Consequently, we propose that G-Rh2 exerts a similar effect by weakening bacterial adhesion and aggregation, thereby enhancing antibiotic penetration into biofilms.

To further clarify the molecular basis of the synergistic antibacterial effect of the combination of G-Rh2 and *β*-lactam antibiotics against MRSA, we performed transcriptome analysis complemented by qRT-PCR validation. The principal mechanism of *β*-lactam antibiotic resistance in MRSA is the acquisition of the *mecA* gene on the staphylococcal cassette chromosome mec (SCCmec) mobile element. The expression of *mecA* is primarily regulated by the *mecR1-mecI* inducer-repressor system (6). In the comparisons of the combined treatment or CFX-treated groups with the control group, we observed an upregulation of *β*-lactam resistance genes, such as *mecA*, *mecR1*, and *mecI*. Consistent with previous studies, this upregulation of *β*-lactam resistance genes results from MRSA strain exposure to sub-MIC levels of *β*-lactam antibiotics (39, 40). However, when comparing the combined treatment group to the CFX-treated group, the expression levels of these resistance genes did not show significant differences. Therefore, we conclude that G-Rh2 enhances the sensitivity of MRSA to *β*-lactam antibiotics, but not directly through the downregulation of *β*-lactam resistance genes. TCS is a crucial bacterial signaling system that receives and relays environmental stress signals, such as antibiotics, osmotic pressure, specific signaling molecules, and membrane stress. It detects these signals through histidine kinases and modulates gene expression via response regulators, thereby coordinating bacterial survival, pathogenicity, and drug resistance (41). The KEGG pathway enrichment analysis, which compared the combined treatment group with the CFX-treated group, revealed that the DEGs were significantly enriched in the TCS pathway. Notably, the addition of G-Rh2 downregulates essential components of the TCS, such as *vraR*, *dltA*, *dltB*, *dltC*, and *dltD*. The *vraS* and *vraR* genes constitute the core of the vancomycin-resistance-associated signal transduction system (VraSR), which mediates antimicrobial resistance in *S. aureus*. Exposure to cell wall-targeting antibiotics, such as *β*-lactams, induces VraSR (42), which in turn controls a large VraSR-dependent cell wall stress stimulon (CWSS). Induction of this CWSS can enhance peptidoglycan synthesis by upregulating genes, such as *pbp2*, *murZ,* and *fmtA*, thereby protecting the cell envelope from damage (43). Numerous studies have shown that inactivation of *vraSR* reduces bacterial resistance to *β*-lactam antibiotics (44, 45), while *mecA* expression remains unaffected (46). Thus, inhibitors of *vraS* or *vraR* may resensitize MRSA to *β*-lactam antibiotics. Our data indicate that CFX alone upregulates *vraR*, while the addition of G-Rh2 results in its downregulation. This suggests that G-Rh2 may act as a *vraR* inhibitor, thereby restoring MRSA susceptibility to *β*-lactam antibiotics. Teichoic acids (TAs) are characteristic cell-wall polymers of Gram-positive bacteria, covalently linked to peptidoglycan as wall teichoic acids (WTAs) or anchored in the cytoplasmic membrane as lipoteichoic acids (47). The *dlt* operon, which includes the core genes *dltA*, *dltB*, *dltC*, and *dltD*, mediates the D-alanylation of anionic TAs. This process reduces the net negative charge of the bacterial surface and enhances resistance to cationic antimicrobial peptides (48, 49). A recent study revealed that D-serine can be integrated into WTAs through the *dlt* operon, resulting in a reduction of WTA content and increasing the susceptibility of MRSA to *β*-lactam antibiotics. This finding underscores the role of the *dlt* operon in modulating *β*-lactam sensitivity (50). Thus, the *dlt* operon represents a promising target for reversing drug resistance in Gram-positive pathogens. Leparfait et al. synthesized novel DltA inhibitors and demonstrated *in vitro* that these compounds restore MRSA sensitivity to imipenem (51). Therefore, the downregulation of the core gene of the *dlt* operon by G-Rh2 may be an additional mechanism through which it reverses MRSA resistance.

The formation of bacterial biofilms primarily relies on the quorum sensing (QS) system, with ABC transporters serving as positive regulatory factors in this process. The QS system promotes the formation of biofilms by interacting with ABC transporters (52). In this study, DEGs were significantly enriched in the ABC transporter pathway. Specifically, genes related to the ABC transporter, such as *pstS*, *pstA*, *pstB*, *pstC*, and *phoU*, were significantly downregulated in both the G-Rh2-treated group and the combined treatment group. These genes constitute the core of the *pst* operon, which regulates the phosphorus-specific transport (Pst) system (53). Previous studies have shown that the Pst system is essential for bacterial uptake and utilization of inorganic phosphate and that it contributes to antibiotic resistance, regulation of invasion, colonization, and biofilm formation (54, 55). Mutations in *pstC* and *pstA* can abolish biofilm formation in *Pseudomonas aureofaciens* (53). In *Pseudomonas aeruginosa*, the phosphate transport protein PstS influences biofilm formation by a phosphate-independent mechanism (56). In *Staphylococcus epidermidis*, PhoU2 rather than PhoU1 modulates growth, biofilm formation, oxidative stress response, and drug tolerance (57). Anthraquinone compounds disrupt the Pst system and thereby induce MRSA biofilm disassembly by perturbing phosphate homeostasis (58). Our study revealed that G-Rh2 treatment significantly downregulated key genes of the *pst* operon, providing a molecular explanation for the impact of G-Rh2 on MRSA biofilm.

Our findings demonstrated that G-Rh2 resensitized MRSA to *β*-lactam antibiotics and exhibited synergistic anti-biofilm activity with these antibiotics. The synergistic effect is closely linked to the downregulation of *vraR*, *dlt* operon, and *pst* operon. However, the efficacy, pharmacokinetic profile, and potential toxicity of this combination in a complex physiological environment remain to be assessed. Consequently, future research should focus on validating the effectiveness and safety of this combination therapy in animal models.

## Data Availability

The raw transcriptome sequencing data generated in this study have been deposited in the NCBI Sequence Read Archive (SRA) under the BioProject accession number PRJNA1404664.

## SUPPLEMENTAL MATERIAL

Supplemental material spectrum.00547-26-s0001.docx Table S1; Fig. S1 to S3.

ASM does not own the copyrights to Supplemental Material that may be linked to, or accessed through, an article. The authors have granted ASM a non-exclusive, world-wide license to publish the Supplemental Material files. Please contact the corresponding author directly for reuse.
