# Supplementary material for: Synergistic activity of Ginsenoside Rh2 with β-lactam antibiotics and its mechanism of action against methicillin-resistant Staphylococcus aureus
Source: Microbiol Spectr. 2026 Sep 28;14(11):e00547-26. doi: 10.1128/spectrum.00547-26 (PMC13729095; doi:10.1128/spectrum.00547-26)
Supplement: Supplementary file 1 [file spectrum.00547-26-s0001.docx]

**TABLE S1.** Primer sequences usABLEed for qRT-PCR.

| **Gene** | **Direction** | **Sequence (5’ to 3’)** |
| --- | --- | --- |
| *16S rRNA* | forward | TTATGGTGCTGGGCAAATACA |
|  | Reverse | CACCATGTAAACCACCAGATA |
| *vraR* | forward | CGCGCAGAGTTATATGAAATGCTT |
|  | Reverse | AGTAATATGCGATGCACTAGCAAT |
| *dltA* | forward | ATTACGCCAGTCTGAGTTCG |
|  | Reverse | GCATTGTCCGTAACTTCAGTCG |
| *dltB* | forward | AATTTGGCATGGTATCGAAGTGT |
|  | Reverse | GATGTTTCTTGCGCCAACGTTC |
| *dltC* | forward | CCAGACGTAGAAATTTTTGAAGAAGG |
|  | Reverse | CCCACTCATCTCTATCAAAGTCCA |
| *dltD* | forward | ATCGATAAAGAACGTCGTCAAGC |
|  | Reverse | CCGATGTGTACGGCATCACT |
| *pstS* | forward | ATGGCTCATCAACAGTAGCAC |
|  | Reverse | GCACCTGTACCAGCTTGTCC |
| *pstC* | forward | GCACGACGCATAATAAAACCGAT |
|  | Reverse | ACTAAGCCGGGACTTATAGCAT |
| *phoU* | forward | GGCTTTGCACGACAAACAGT |
|  | Reverse | TACGCAAATCACTCGCAATGGG |
| *mecA* | forward | TGGCAGACAAATTGGGTGGT |
|  | Reverse | TGAAGCAACCATCGTTACGGA |
| *mecR1* | forward | GTGCTCGTCTCCACGTTAATTCCA |
|  | Reverse | GACTAACCGAAGAAGTCGTGTCAG |

**FIG S1.** KEGG enrichment scatter plots for DEGs resulting from the control group and the G-Rh2-treated group.

**FIG S2.** KEGG enrichment scatter plots for DEGs resulting from the control group and the CFX-treated group.

**FIG S3.** KEGG enrichment scatter plots for DEGs resulting from the control group and the combined treatment group.
